# Enhancement of Arterial Pressure Pulsatility by Controlling Continuous-Flow Left Ventricular Assist Device Flow Rate in Mock Circulatory System

**DOI:** 10.1007/s40846-016-0140-1

**Published:** 2016-06-25

**Authors:** Selim Bozkurt, Frans N. van de Vosse, Marcel C. M. Rutten

**Affiliations:** Department of Biomedical Engineering, Eindhoven University of Technology, PO Box 513, GEM-Z 4.18, 5600 MB Eindhoven, The Netherlands

**Keywords:** Continuous-flow left ventricular assist device (CF-LVAD), Arterial pulsatility, In vitro experiment

## Abstract

Continuous-flow left ventricular assist devices (CF-LVADs) generally operate at a constant speed, which reduces pulsatility in the arteries and may lead to complications such as functional changes in the vascular system, gastrointestinal bleeding, or both. The purpose of this study is to increase the arterial pulse pressure and pulsatility by controlling the CF-LVAD flow rate. A MicroMed DeBakey pump was used as the CF-LVAD. A model simulating the flow rate through the aortic valve was used as a reference model to drive the pump. A mock circulation containing two synchronized servomotor-operated piston pumps acting as left and right ventricles was used as a circulatory system. Proportional-integral control was used as the control method. First, the CF-LVAD was operated at a constant speed. With pulsatile-speed CF-LVAD assistance, the pump was driven such that the same mean pump output was generated. Continuous and pulsatile-speed CF-LVAD assistance provided the same mean arterial pressure and flow rate, while the index of pulsatility increased significantly for both arterial pressure and pump flow rate signals under pulsatile speed pump support. This study shows the possibility of improving the pulsatility of CF-LVAD support by regulating pump speed over a cardiac cycle without reducing the overall level of support.

## Introduction

Continuous-flow left ventricular assist devices (CF-LVADs) generally operate at a constant speed. However, constant-speed CF-LVAD assistance reduces the pulse pressure and index of pulsatility over a cardiac cycle, which may lead to long-term complications [1]. Patients under pulsatile support exhibit less remodeling and functional changes in their vascular system compared to those of patients under constant-flow support [2–9]. This leads to less gastrointestinal (GI) bleeding, aortic wall remodeling, and better vascular auto-regulatory function. Geisen et al. reported that non-surgical bleeding in CF-LVAD patients can be explained by acquired von Willebrand disease [10]. However, Crow et al. reported that loss of von Willebrand factor multimeres alone cannot be a predictor of GI bleeding [11]. Nevertheless, comparative studies showed that loss of von Willebrand factor is higher under CF-LVAD support than under pulsatile assist device support [12, 13], which may be interpreted as pulsatile circulatory support being more beneficial for CF-LVAD patients. Under pulsatile support, pulmonary vascular resistance reduces more than it does under constant flow support [14]. Furthermore, long-term organ function appears to be preserved better with pulsatile support [15–17]. Inflammatory responses reportedly occur at a lower rate in patients under pulsatile support as well [18, 19].

Comparison results of pulsatile vs. continuous flow cardiac support and the benefits of pulsatile perfusion have been summarized in the literature [20–22]. From these studies, it is clear that pulsatile support may be beneficial for reducing the late complications of CF-LVAD support. Furthermore, solutions have been proposed to increase arterial pulsatility. For instance, Shiose et al. [23] proposed a speed modulation algorithm for a continuous-flow total artificial heart to generate physiologic arterial waveforms. They generated three speed profiles, namely sinusoidal, rectangular, and optimized (physiological) speed profiles, and assessed them using a mock circulatory system. Arterial pulse pressure was highest under the rectangular speed profile of CF-LVAD. A stepwise CF-LVAD operating speed change over a cardiac cycle was proposed to increase arterial pulsatility [24] by changing the pump speed from a low value to a high value at the peak systole to keep the arterial pressure increasing despite the start of left ventricle relaxation. CF-LVAD operating speed was reduced to a low value again at the end of the systole to reduce the CF-LVAD output at the diastolic phase. Although such a CF-LVAD driving mode increases arterial pulsatility, its implementation was not considered in this particular study. A similar method for adjusting CF-LVAD operating speed at high and low levels over a cardiac cycle has been proposed [25]. Unlike the preceding application, the high CF-LVAD operating speed was applied over the entire systolic phase in this study. A method for synchronizing the CF-LVAD speed and systolic phase has been proposed [26]. A pacing lead was used to detect the ventricular electrocardiogram for the synchronization of the RBP speed change. The energy equivalent pressure was 9 % higher than the mean aortic pressure under pulsatile-speed CF-LVAD support when the CF-LVAD was operated in co-pulsative mode, whereas these two parameters were almost at the same level under constant-speed CF-LVAD support. Shi et al. [27, 28] evaluated the cardiovascular response under pulsatile CF-LVAD support using a numerical model by applying various pulsation ratios and phase shift values to the RBP motion profile. An optimization algorithm for balancing the importance of the characteristic cardiovascular variables by introducing a cost function was used. In these studies, the operating speed of the CF-LVAD was changed directly. Although this increases the arterial pulsatility, it may not generate physiological pressure and flow signals in the arteries. Using CF-LVAD flow rate signals rather than direct speed control would generate more physiological blood flow in the arteries. Such an application was presented [29, 30] by using various CF-LVAD flow rate signal profiles to modulate the operating speed in order to increase pulsatility. The pulse width and amplitude of the flow signal through the pump was changed to assess the effects of the CF-LVAD flow profiles. Although the possibility of increasing pulsatility through pump control was shown, the control method itself was not considered in the numerical simulations. Therefore, this study was unclear in terms of control system design and whether such an operation mode for CF-LVAD is achievable for flow rate signals with relatively high amplitude.

The present study aims to operate a CF-LVAD in co-pulsation support mode, similar to previous studies [31, 32], and assess whether the co-pulsation support mode significantly increases arterial pulsatility in a mock circulatory system. Furthermore, a complete control method is presented and applied experimentally by using a physiological model to describe the reference pump flow. Flow rate was used as a control variable instead of pump operating speed, unlike in other studies.

## Materials and Methods

**Fig. 1:**
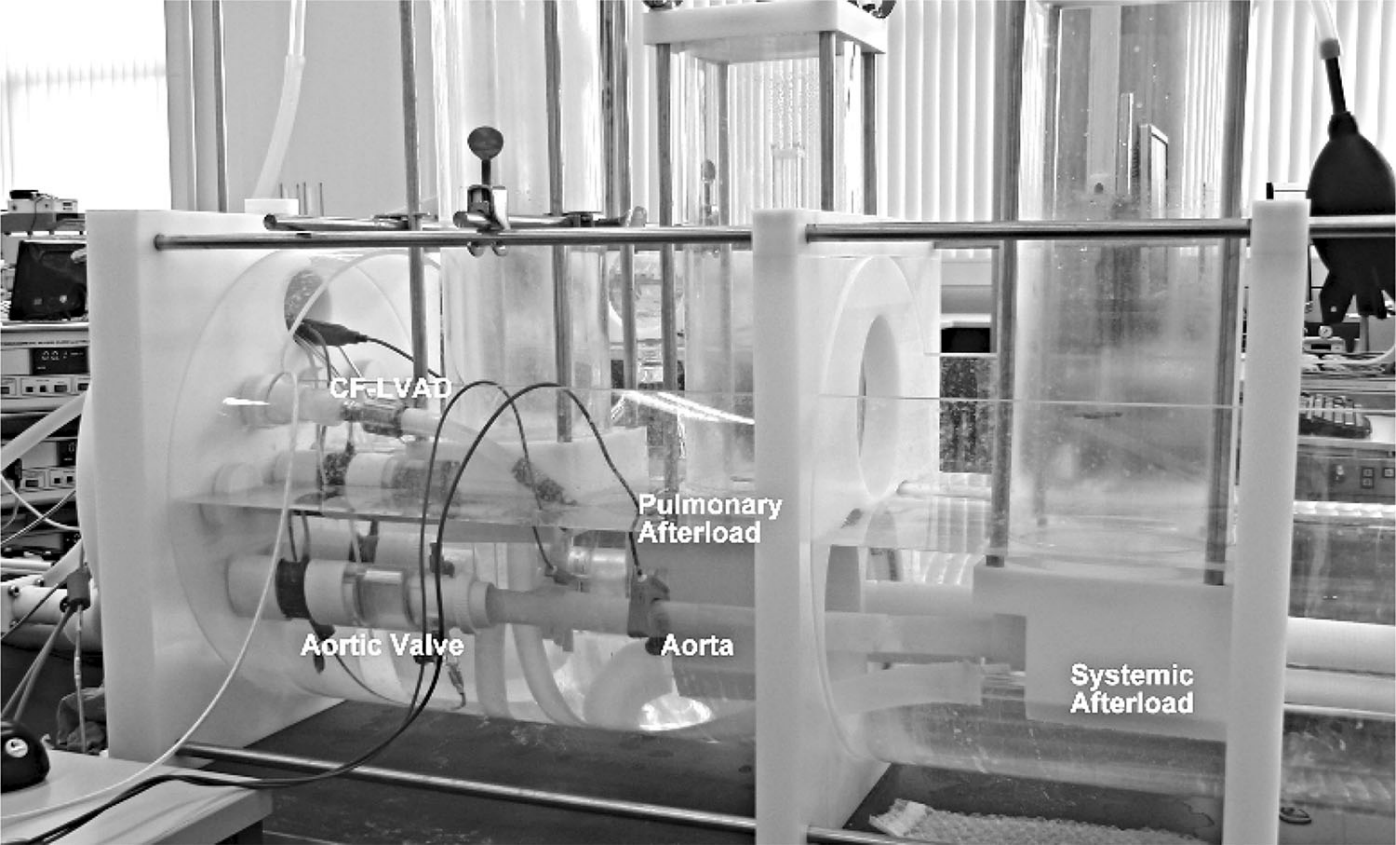
Mock circulation system used in experiments

The mock circulatory system, shown in Fig. 1, contained two synchronized servomotor-operated piston pumps acting as the left and right ventricles, respectively. The ventricles were connected to the circulation system via two polyurethane valves. The systemic circulation was modeled by a cylindrical, polyurethane tube with a constant diameter of 25 mm. The systemic and pulmonary impedance were modeled by four-element Windkessel models, positioned distal from the aorta and pulmonary valve, respectively. Compliance chambers represented passive atria and functioned as a preload for ventricular filling. Further information about this system can be found elsewhere [33].

A numerical model describing the left atrial, left ventricular, and systemic arterial dynamics was used for deriving the reference flow rate over a cardiac cycle in the control application. The ventricle model is based on the model developed by Bovendeerd et al. [34]. This model describes the ventricular wall mechanics using myocardial constitutive properties. The ventricular wall mechanics model relates the macroscopic ventricular pressure and volume to microscopic tissue properties, namely fiber stress, fiber strain, radial wall stress, and radial wall strain. Active and passive fiber stress relations include the myocardial constitutive laws for fiber stress and radial stress. Detailed information about the full heart model can be found elsewhere [34]. The circulatory system is described with a lumped parameter model including electrical analogues for resistance and compliance [35]. Similarly, the heart valves were modeled as ideal diodes, allowing one-way blood flow. The left atrium was modeled as a passive compliance only.

To regulate the CF-LVAD operating speed, straightforward proportional-integral (PI) control was applied as the control method. The control application includes three main components, namely a PI controller, a DC motor driver, and the CF-LVAD. A PI controller is defined as:

1 $$ u\left( t \right) = K_{p} \cdot e\left( t \right) + K_{i} \int\limits_{0}^{t} {e\left( \theta \right)} d\theta $$

**Fig. 2:**
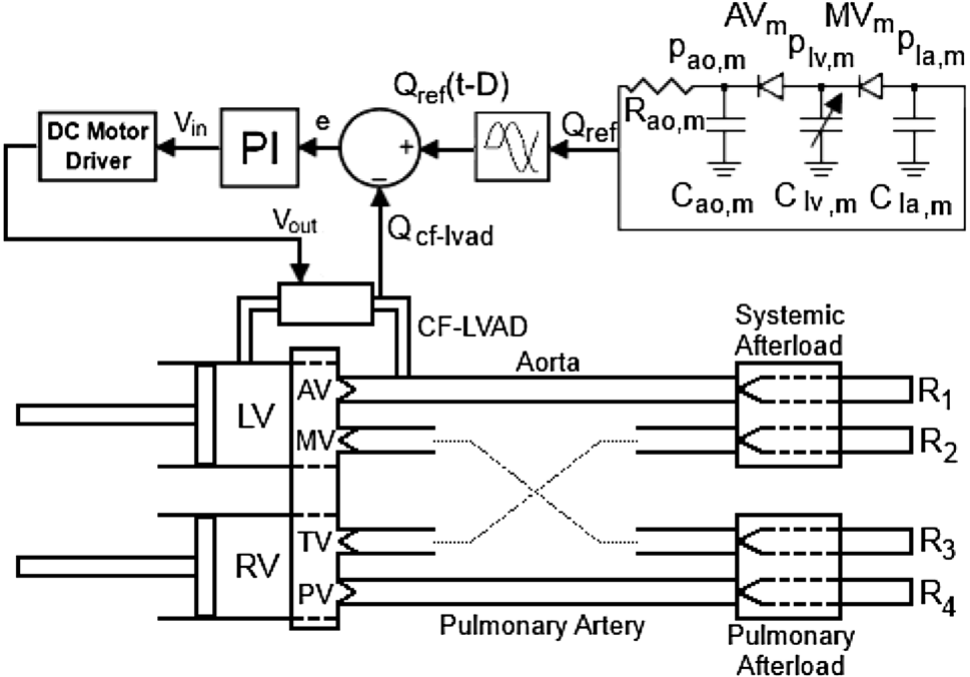
Schematic representation of mock circulation (*top view*) and block diagram of control application. *Q* and *p* are flow rate and pressure, LV and RV represent the left and right ventricles, *R* and *C* denote resistance and compliance, respectively. *MV*, *AV*, *TV*, and *PV* are mitral, aortic, tricuspid, and pulmonary valves, and *ao*, *lv*, and *la* are aorta, left ventricle, and left atrium, respectively. *m* is model, *ref* is reference, and *e*, *V*_*in*_, and *V*_*out*_ are error and input and output voltages of DC motor driver in the control application, respectively. *D* is applied delay to synchronize CF-LVAD flow rate, and *1*, *2*, *3*, and *4* denote resistances in afterload sections

In a PI control application, proportional gain (*K*_*p*_) is multiplied by the control error *e*(*t*) to reduce the instant difference between the reference CF-LVAD flow rate and actual CF-LVAD flow rate. Integral gain (*K*_*i*_) is multiplied by the integration of the control error over time. The sum of both actions (P and I) constitutes the output of the controller, *u*(*t*), which is the input voltage (*V*_*in*_) of the DC motor driver in this application. The output of the DC motor driver (*V*_*out*_) was the input of the CF-LVAD motor, which drives the impeller in the heart pump. A schematic representation of the mock circulation and the block diagram of the control application are given in Fig. 2.

The control algorithm was developed using Matlab Simulink R2006a and employed a Runge–Kutta solver scheme. The time step was 0.001 s. To enable pulsatile pump speed control, an industrial brushless DC motor driver was used (Maxon DECS 50/5, Maxon, Switzerland) for driving the CF-LVAD. This driver uses an input voltage as the reference signal to regulate the pump operating speed towards a certain level. The maximum signal input voltage of this driver is 5 V [36]. A MicroMed DeBakey CF-LVAD was used as the assist device. Compared to other devices, this device is more responsive to the control input signals over the duration of a cardiac cycle. Additionally, it has a flow sensor attached, enabling direct feedback of the control variable. In the control application, the peak of the CF-LVAD flow was applied at peak systole to maximize the peak arterial pressure and arterial pulse pressure. However, this device cannot respond fast enough to the control input signals. Therefore, there is a delay between the reference CF-LVAD flow and actual CF-LVAD flow. The duration of this delay (*D*) is the time elapsed between the peak of the reference CF-LVAD flow and the peak of the actual CF-LVAD flow (Fig. 5). Therefore, the peak of the reference CF-LVAD flow signal should be applied before the peak systole, considering the duration of this delay (*D*), to apply the peak of the actual CF-LVAD flow at the peak systole. The reference CF-LVAD flow rate signal was shifted to the left on the time axis by using a delay block from the Matlab Simulink library (Fig. 2) in the control algorithm. Furthermore, the mock circulatory system is controlled by using Matlab Simulink R2006a. It was thus possible to acquire the pressure, flow data, and time over a cardiac cycle and synchronize the control signals with the mock circulatory system.

In the experiments, left ventricular and aortic pressures were monitored using pressure sensors (Becton–Dickinson Medical P10EZ-1). The CF-LVAD flow rate was measured with a Transonic ME13PXN flow probe at the outlet of the pump, connected to a Transonic 410 flow meter (Transonic, Ithaca, NY).

The pulsatility was quantified using the index of pulsatility (*I*_*p*_) [37] defined as:

2 $$ I_{p} = {{\left( {X_{\hbox{max} } - X_{\hbox{min} } } \right)} \mathord{\left/ {\vphantom {{\left( {X_{\hbox{max} } - X_{\hbox{min} } } \right)} {X_{\text{mean}} }}} \right. \kern-0pt} {X_{\text{mean}} }} $$

where *X* denotes the hemodynamic variable that is considered in the calculation of the index of pulsatility.

For comparison, the CF-LVAD was operated at a constant speed, generating the same mean pump output as that obtained in pulsatile pump speed control mode.

The data obtained from the experiments were analyzed using one tailed *t* test in Microsoft Excel 2007. A *p* value of less than 0.05 was considered to be significant.

First, the experiments were performed under healthy and pathological conditions. The contractility of the left ventricular model in the mock circulatory system was reduced in order to obtain the dilated cardiomyopathy (DCM) conditions. A total of ten experiments were performed for the assisted circulatory system with constant- and pulsatile-speed CF-LVAD assistance. The heart rate was kept at 75 bpm during the experiments. The systemic arterial resistance was adjusted manually to obtain physiological pressure and flow signals in the systemic arteries. Contractility values that represented mild (*c* = 0.8) and severe (*c* = 0.5) heart failure were used to assess the pump performance and improvement in pulsatility under pulsatile-speed CF-LVAD support. The settings applied in the experiments for the assisted circulation are given in Table 1.

**Table 1:** Applied settings in experiments for assisted circulation

	Contractility	Reference CF-LVAD flow rate (L/min)
1	0.5	2.6
2	0.5	3.1
3	0.5	3.6
4	0.5	4.1
5	0.5	4.6
6	0.8	2.5
7	0.8	3.0
8	0.8	3.9
9	0.8	4.5
10	0.8	5.0

## Results

**Fig. 3:**
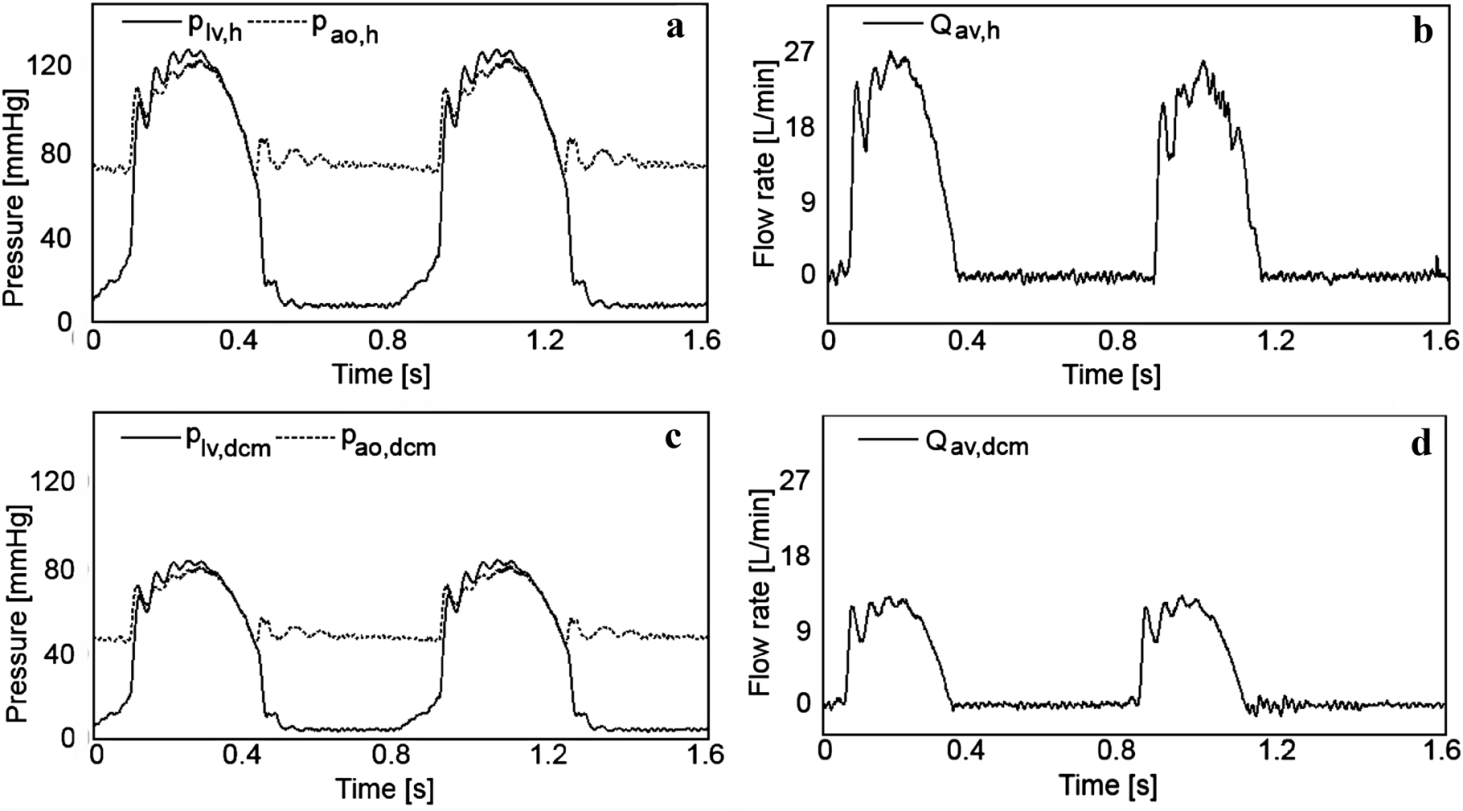
Experimental results for healthy (*h*) and DCM (*dcm*) conditions. **a** *p*_*lv,h*_ and *p*_*ao,h*_ are pressures in left ventricle and aorta, respectively, for healthy settings, **b** *Q*_*av,h*_ is flow rate through aortic valve for healthy settings, **c** *p*_*lv,DCM*_ and *p*_*ao,DCM*_ are pressures in left ventricle and aorta, respectively, for DCM settings, and **d** *Q*_*av,DCM*_ is flow rate through aortic valve for DCM settings

Representative left ventricular and aortic pressure signals and the flow rate signal through the aortic valve for healthy and DCM conditions are given in Fig. 3.

**Fig. 4:**
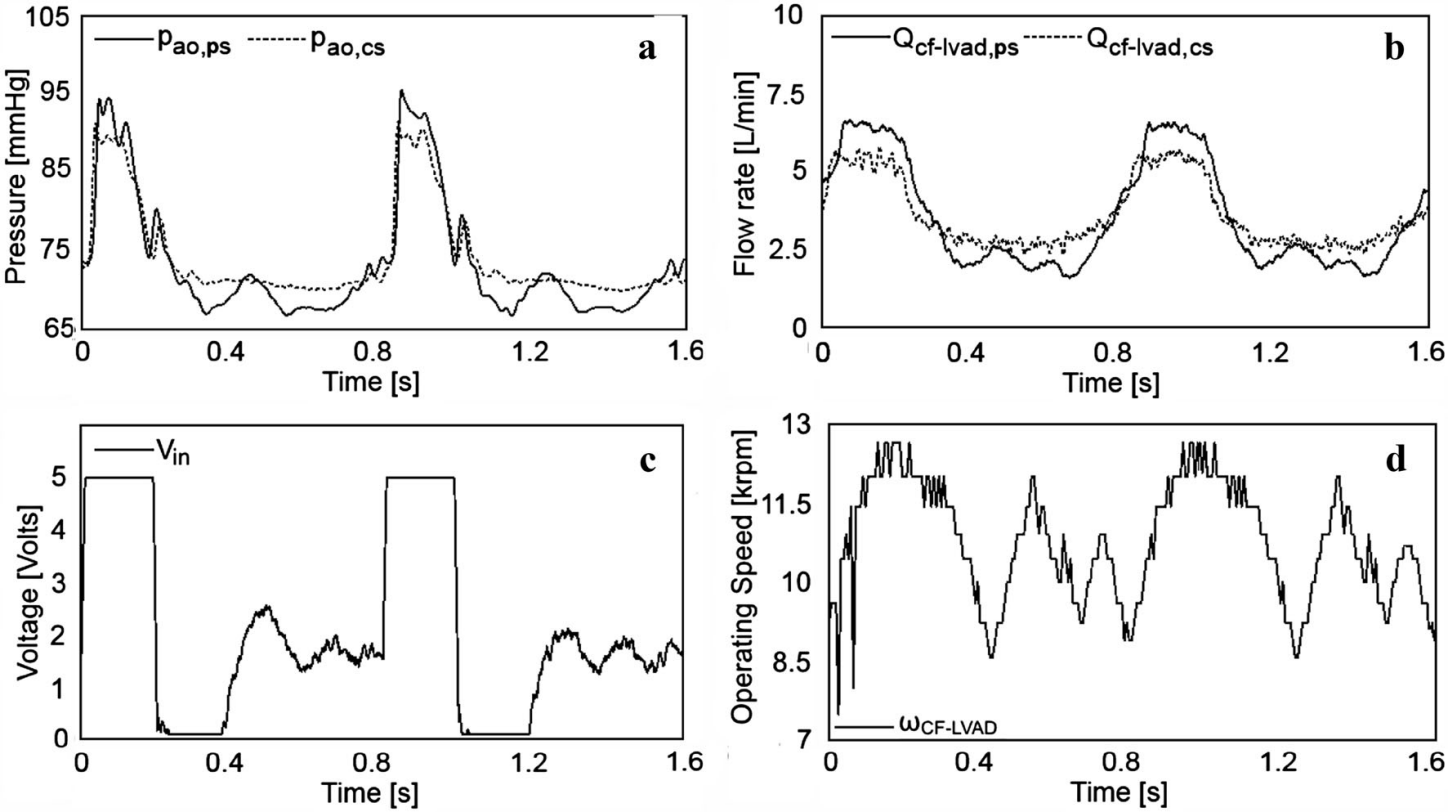
**a** Aortic pressures (*p*_*ao*_) under pulsatile- (*ps*) and constant-speed (*cs*) CF-LVAD operation modes, **b** pump flow rates (*Q*_*cf*-*lvad*_) under pulsatile- (*ps*) and constant-speed (*cs*) CF-LVAD operation modes, **c** DC motor driver system input voltage (*V*_*in*_), and **d** CF-LVAD operating speed (*ω*_CF_LVAD_)

The results are given for the CF-LVAD-assisted failing heart under various assistance modes for a given mean pump output (3.6 L/min). The aortic pressure and pump flow rate under pulsatile- and constant-speed CF-LVAD assistance modes are given in Fig. 4a, b, respectively. The CF-LVAD input voltage and operating speed for the pulsatile-speed pump support mode are given in Fig. 4c, d, respectively.

**Fig. 5:**
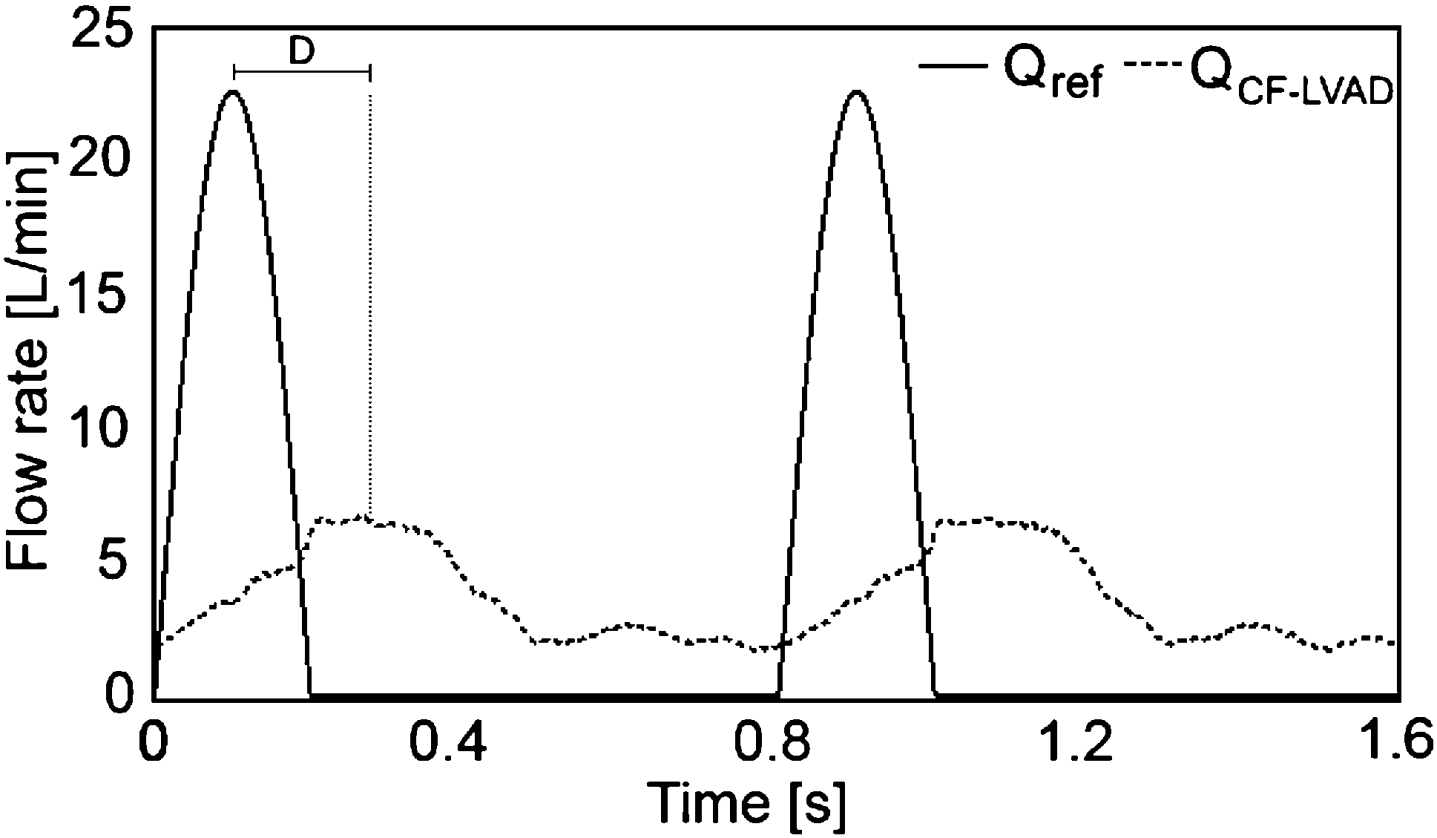
Reference CF-LVAD flow rate (*Q*_*ref*_) and actual CF-LVAD flow rate (*Q*_*CF*-*LVAD*_) (*D* represents applied delay synchronizing CF-LVAD flow rate)

Under pulsatile-speed CF-LVAD assistance mode, the amplitudes of the aortic pressure signals are larger than those obtained with constant-speed CF-LVAD assistance. Under both operation modes, the mean aortic pressure was 86 mmHg. Under pulsatile-speed CF-LVAD support, the amplitude of the measured pump flow signals was higher than that of the flow signal with constant-speed CF-LVAD support. The mean flow rate was 3.6 L/min for both operation modes. A sudden increase in the CF-LVAD input voltage was observed due to the applied reference pump flow in the systolic phase. The input voltage saturated at 5 V because of the DC motor driver limits. A rapid decrease followed the saturation with the relaxation of the ventricle and the input voltage decreased to a minimum. The input voltage increased again in the diastolic phase to avoid regurgitant CF-LVAD flow due to a relatively high afterload. The CF-LVAD operating speed shows a similar change over a cardiac cycle. It increased to a maximum level at around 12.5 k rpm in the systolic phase and decreased to around 8.5 k rpm with the relaxation of the left ventricle. It increased in the diastolic phase due to a high afterload to avoid reverse flow through the pump. The reference and measured CF-LVAD flow rates are given in Fig. 5.

There was a delay (*D*) between the reference and measured CF-LVAD flow signals in pulsatile-speed operation mode. Although the CF-LVAD input voltage and operating speed increased in the diastolic phase as well as the systolic phase, there was no significant increase in the CF-LVAD flow because of the high afterload. Therefore, increases in the input voltage and operating speed of the CF-LVAD slightly affected the pump flow.

**Fig. 6:**
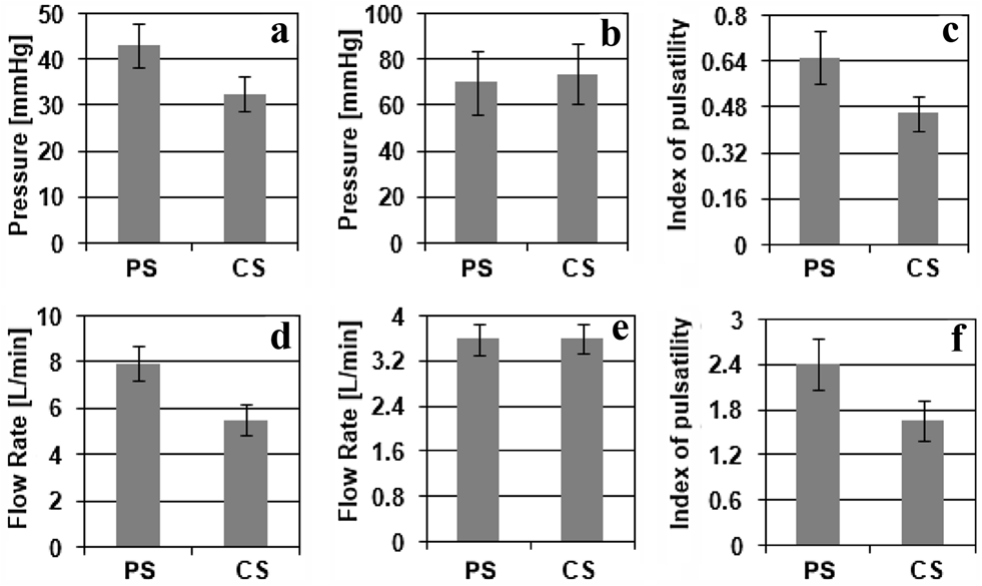
**a** Arterial pulse pressure, **b** mean aortic pressure, **c** index of pulsatility in aortic pressure, **d** amplitude of CF-LVAD flow rate, **e** mean pump output, and **f** index of pulsatility in pump flow rate under constant- (*CS*) and pulsatile-speed (*PS*) CF-LVAD support (*error* *bars* represent 2× standard deviation)

Figure 6 shows a comparison of the hemodynamic variables under assistance with various operation modes. Under constant-speed CF-LVAD assistance, the aortic pulse pressure was lower than that under pulsatile-speed CF-LVAD operation mode (*p* < 0.05). For the two operation modes, the mean aortic pressure was at the same level (*p* > 0.05). Similar to the aortic pulse pressure, the index of pulsatility in the aortic pressure was significantly higher under pulsatile-speed CF-LVAD support (*p* < 0.05). Also, the generated CF-LVAD flow rate was the same in both support modes (*p* > 0.05), while the amplitudes of the CF-LVAD flow rate and index of pulsatility were both significantly higher under pulsatile-speed CF-LVAD support (*p* < 0.05).

## Discussion

The aim of this study was to show the possibilities of pulsatile CF-LVAD assistance compared to constant-speed support in a mock circulatory system. The operating speed of a MicroMed DeBakey CF-LVAD was regulated by applying PI control to the pump flow rate to increase arterial pulsatility. The proposed control strategy increased the pulse pressure and *I*_*p*_ in the arterial pressure signals over a cardiac cycle without reducing the level of support. The applied control strategy was used to increase the pump flow at the systolic phase and to minimize flow in the diastolic phase. This strategy increased the arterial pulsatility index by regulating the pump operating speed. However, a change in arterial pulsatility depends on several factors, such as the preload and afterload of the left ventricle and the pressure-flow relation at different speeds of the CF-LVAD. With a change in the preload and afterload, the pulsatility index increases due to synchronization of the pump and the heart. The level of increase is determined by the pressure-flow characteristics of the CF-LVAD.

The flow rate through the CF-LVAD did not strictly follow the reference model due to the design of the pump. The main mechanical components of the CF-LVAD are the inducer, the impeller, and the diffuser. The main electrical components are the stator and rotor, which also functions as the impeller. It is thought that because of the gap between the embedded magnets in the impeller and the coil in the stator, the pump response is not sufficiently fast to follow the reference. Moreover, the DC motor allows a 5-V maximum input voltage to drive the pump. Using feedback control increased arterial pulsatility (Fig. 4a). The flow sensor Micromed CF-LVAD allows the flow rate through the pump to be measured, which makes the operation mode reliable even during long-term use. Although the instantaneous tracking error was considerable in the pulsatile-speed CF-LVAD support mode, the control algorithm doubled the amplitude and pulsatility index in arterial pressure and the CF-LVAD flow rate signals. Due to the construction of CF-LVADs, fast changes of pump speed are difficult to achieve. Adding a more aggressive pump speed control may be convenient to increase the benefit of the applied control strategy. This, however, will lead to higher power consumption, which, in view of the battery power current CF-LVADs rely on, may be difficult to achieve.

The strength of the contractions in the heart is an important parameter affecting the pulsatility in the arteries. The heart becomes more dominant for the higher contractility values. Therefore, the pulsatile-speed CF-LVAD generates less pulsatility in comparison to lower contractility values. Nevertheless, speed variations in both heart failure modes increase arterial pulsatility.

It has been shown that co-pulsative CF-LVAD support increases arterial pulsatility in animal experiments and isolated beating heart experiments [26, 32]. However, direct rotation speed control does not yield physiological shapes in the arterial pressure and flow signals [26]. An approach [32] that applies flow rate control to regulate the CF-LVAD operating speed accordingly over a cardiac cycle has been presented. In that study the applied reference model was able to generate physiological pressure and flow signals and increase arterial pulsatility and it was described by a few parameters. In this application, the parameters can be adjusted to generate a reference model for the changing conditions in a patient.

Synchronization of the CF-LVAD speed is important to achieve such an operation mode in patients. Most CF-LVAD patients have an implantable cardioverter defibrillator along with a CF-LVAD [38]. In these patients, synchronization of the CF-LVAD can be done according to the cardioverter defibrillator signals. Also, natural ECG has a very sharp peak (the R-peak in the QRS-complex) at the beginning of the heart cycle. This peak is widely used for synchronization purposes clinically, e.g., in imaging methods such as magnetic resonance imaging or computed tomography. Synchronizing a pump with this signal should not be difficult. Ando et al. presented such an example in their study [26].

## Conclusion

Numerous studies have reported the beneficial effects of pulsatile mechanical circulatory support over continuous-speed support. This study showed and quantified the effects of pulsatile operating speed over a cardiac cycle to improve arterial pulsatility. Statistical analysis showed that there is a significant increase in arterial pulsatility under co-pulsative CF-LVAD support mode.
